# Microglia-Mediated Ependymal Injury in Bacille Calmette-Guérin-Induced Meningitis Is Attenuated by Sodium Butyrate with Restoration of Hmgcs2 Expression

**DOI:** 10.3390/arm94040054

**Published:** 2026-07-27

**Authors:** Yang Ren, Danni Chen, Shiqi Xie, Yawen He, Xuanru Zhuang, Dan Ye, Zhentao Fei, Lu Xia, Yongjie Wang, Feng Li

**Affiliations:** 1 Department of Pulmonary and Critical Care Medicine, Shanghai Public Health Clinical Center, Fudan University, Shanghai 201508, China; renyang@shaphc.org (Y.R.); xieshiqi_17@163.com (S.X.); 24211300005@m.fudan.edu.cn (Y.H.); 25211300005@m.fudan.edu.cn (X.Z.); 2 School of Pharmacy, Hangzhou Normal University, Hangzhou 311121, China; chendanni0236@163.com; 3 Tuberculosis Research Center, Shanghai Public Health Clinical Center, Fudan University, Shanghai 201508, China; yedan@shaphc.org (D.Y.); feizhentao@shaphc.org (Z.F.); xialu@shaphc.org (L.X.)

**Keywords:** Tuberculous meningitis, ependymal cells, neuroinflammation, sodium butyrate, Hmgcs2, transcriptional regulation

## Abstract

**Highlights:**

**What are the main findings?**

- Microglia amplify ependymal cell transcriptional dysregulation through paracrine signaling in tuberculous meningitis (TBM), with Hmgcs2 identified as a core target regulating ependymal ciliary function and apoptosis.
- Sodium butyrate protects against BCG-induced ependymal injury by upregulating Hmgcs2 expression.

**What are the implications of the main findings?**

- The microglia–ependymal cell crosstalk via Hmgcs2 pathway provides a novel mechanistic understanding of TBM pathogenesis.
- Hmgcs2 and its regulation by sodium butyrate represent a potential therapeutic target for mitigating ependymal injury in TBM.

**Abstract:**

Background: Tuberculous meningitis (TBM) is the most severe form of central nervous system tuberculosis, associated with high mortality and neurological sequelae. Microglia-driven neuroinflammation is a key contributor to TBM pathogenesis; however, its specific effects on ependymal cells—critical for cerebrospinal fluid dynamics and barrier function—and potential therapeutic strategies remain unclear. Methods: A murine TBM model was established by tail vein injection of BCG. Although the virulence of BCG, an attenuated strain of Mycobacterium bovis, is different from that of clinically isolated human Mycobacterium tuberculosis, its induced phenotypes such as periventricular inflammatory infiltration, microglia activation, and ependymal dysfunction highly reproduce the key histopathological features of human TBM. Primary ependymal cells were cultured and treated either directly with BCG or indirectly with conditioned medium from BCG-stimulated BV2 microglial cells (BCG+BV2-CM). Transcriptomic profiling was conducted via RNA sequencing, with validation by qPCR and Western blot. Functional outcomes, including ciliary morphology and apoptosis, were assessed using immunofluorescence and flow cytometry. The therapeutic effect of sodium butyrate (NaB) was evaluated through pretreatment experiments. Results: BCG infection induced characteristic TBM pathology, with persistent bacteria in the brain and lungs, ventricular inflammation, and pulmonary damage. Transcriptomic analysis showed that direct BCG treatment altered the expression of 1036 genes in ependymal cells, whereas BCG+BV2-CM treatment induced 3558 differentially expressed genes, highlighting microglia’s role in amplifying ependymal injury. Integrated analysis identified 64 consistently dysregulated genes across in vitro and in vivo models, enriched in immune and metabolic pathways. BCG challenge significantly downregulated Hmgcs2, leading to ciliary shortening and increased apoptosis. Sodium butyrate treatment restored Hmgcs2 expression, preserved ciliary structure, and reduced apoptosis. Conclusion: Microglia profoundly exacerbate transcriptional dysregulation in ependymal cells during TBM. Sodium butyrate confers protection against BCG-induced ependymal damage by upregulating Hmgcs2, revealing a novel therapeutic target for tuberculous meningitis.

## 1. Introduction

Tuberculous meningitis (TBM) represents a paradigm of infection-induced neuroimmunopathology, in which dysregulated host immune responses paradoxically exacerbate neuronal injury and disease progression [1,2]. Although antimicrobial therapy effectively targets Mycobacterium tuberculosis, it remains inadequate in controlling the maladaptive neuroinflammation that underlies TBM’s devastating neurological sequelae [3,4]. This therapeutic limitation highlights the urgent need to elucidate cell-type-specific mechanisms and intercellular signaling events that perpetuate inflammatory cascades within the central nervous system (CNS) [2,5,6].

Ependymal cells, once regarded primarily as a structural barrier, are now recognized as pivotal regulators of CNS homeostasis. These multiciliated epithelial cells coordinate cerebrospinal fluid (CSF) flow through rhythmic ciliary beating, maintain blood–CSF barrier integrity, and facilitate selective molecular exchange between fluid compartments [7,8,9]. Their strategic location at the brain–CSF interface makes them exceptionally vulnerable to inflammatory insults, implicating them as potential sentinels and amplifiers of neuroinflammation [10,11]. Ependymal cell dysfunction has been mechanistically linked to hydrocephalus, impaired glymphatic clearance, and disrupted neurogenesis [12,13,14]. Nevertheless, their role during infectious conditions—particularly their intrinsic responses to pathogenic challenge—remains a fundamental and unresolved question in neuroimmunology [15,16].

Our previous single-cell RNA sequencing study in a BCG-induced murine model of TBM demonstrated that ependymal cells undergo the most pronounced transcriptional reprogramming among all CNS cell types, with significant alterations in genes involved in metal ion homeostasis, vesicular trafficking, and ciliary function [17,18]. These observations prompt several key mechanistic questions: Does BCG directly induce ependymal cell dysfunction, or is its effect primarily mediated through microglial activation? What are the specific molecular pathways by which BCG triggers transcriptional dysregulation in ependymal cells? Furthermore, which intracellular signaling cascades integrate these pathogenic signals to drive functional deficits? Most importantly, can this injury process be targeted therapeutically to preserve ependymal barrier integrity and mitigate neurological damage [7,8,19]?

In this study, we employ integrated transcriptomic and functional analyses to dissect the cell-intrinsic mechanisms of ependymal injury elicited by direct BCG exposure. We delineate the specific transcriptional programs activated in ependymal cells following BCG challenge, identify Hmgcs2—a rate-limiting enzyme in ketogenesis [20,21,22]—as a crucial mediator of ependymal resilience that is suppressed by BCG, and demonstrate that sodium butyrate (NaB) [23,24,25] restores structural and functional integrity via the epigenetic enhancement of Hmgcs2 expression. Our results uncover a previously unrecognized metabolic axis at the brain–CSF interface and offer a mechanistic rationale for novel therapeutic strategies in TBM.

## 2. Materials and Methods

### 2.1. Animal Model and Ethical Considerations

Female C57BL/6 mice (6 weeks old, weighing 18–22 g) were purchased from Shanghai SLAC Laboratory Animal Co., Ltd. (Shanghai, China). Mice were housed under specific pathogen-free conditions with controlled temperature (22 ± 1 °C) and humidity (55 ± 5%) and a 12/12 h light/dark cycle. To establish the tuberculous meningitis (TBM) model, mice were infected via tail vein injection with Mycobacterium bovis (BCG, YC263066, Shanghai, China), 1 × 10^4^ CFU in 100 μL phosphate-buffered saline (PBS, HyClone, Logan, UT, USA). Control animals received an equal volume of sterile PBS. Mice were euthanized at predetermined time points for tissue collection. All animal experiments were approved by the Institutional Animal Care and Use Committee (IACUC) of the Shanghai Public Health Clinical Center. To ensure model consistency, all infected mice received the same dose of BCG and were not subdivided into subgroups according to clinical phenotype. Animal grouping was performed in a randomized block design and was matched with body weight and baseline status as covariates.

### 2.2. Histopathological Analysis

Brain and lung tissues were harvested and fixed in 4% paraformaldehyde (Biosharp, Hefei, China), embedded in paraffin, and sectioned. Tissue sections were stained with hematoxylin (Sigma-Aldrich, St. Louis, MO, USA) and eosin (Sigma-Aldrich) using standard protocols to assess inflammatory infiltration and pathological changes. Images were captured using a light microscope (Nikon, Tokyo, Japan).

### 2.3. Primary Murine Ependymal Cell Culture and Differentiation

Primary murine ependymal cells (mEPCs) were isolated and differentiated as previously described, with modifications [12,26]. Briefly, cerebral ventricular tissues were dissected from postnatal day 1–3 C57BL/6 mouse pups under aseptic conditions. Tissue fragments were digested enzymatically in DMEM (Gibco, Grand Island, NY, USA) containing papain (2 mg/mL; Worthington Biochemical, Lakewood, NJ, USA), DNase I (0.15 mg/mL; Sigma-Aldrich), L-cysteine (0.3 mg/mL; Sigma-Aldrich), and 1% penicillin/streptomycin (Beyotime, Shanghai, China) for 30 min at 37 °C. Digestion was terminated using L15 medium (Sigma-Aldrich) supplemented with trypsin inhibitor (1 mg/mL; Sigma-Aldrich), DNase I (0.2 mg/mL; Sigma-Aldrich), and 10% fetal bovine serum (FBS; Gibco). Cells were pelleted, resuspended, and seeded into poly-L-lysine-coated (Sigma-Aldrich) T25 flasks in DMEM complete medium (10% FBS, 1% P/S). On day 7, confluent cultures were shaken at 100 rpm for 4 h to remove loosely adherent neurons, then trypsinized (Trypsin-EDTA; Beyotime) and replated onto poly-L-lysine-coated coverslips at a density of 2 × 10^5^ cells per well. Serum-free DMEM was applied 24 h post-plating to induce ependymal differentiation (designated as D0). The medium was changed every 3–4 days. Successful differentiation and cilia formation of mEPCs were confirmed by immunofluorescence co-staining for acetylated-α-tubulin (antibody from Abcam, Cambridge, UK) and DAPI (Sigma-Aldrich), which revealed characteristic multiciliated structures.

### 2.4. Microglial Cell Culture and Conditioned Medium Preparation

The BV2 murine microglial cell line was obtained from the Cell Bank of the Chinese Academy of Sciences (Shanghai, China) and cultured in DMEM (Gibco) supplemented with 10% FBS (Gibco) and 1% penicillin–streptomycin (Beyotime) under standard conditions (37 °C, 5% CO_2_). For conditioned medium preparation, cells were seeded at 5 × 10^5^ cells/well in 6-well plates. Upon reaching 70–80% confluence, cells were stimulated with BCG at a multiplicity of infection (MOI) of 10 for 24 h. Control cultures received equivalent volumes of PBS (HyClone). Conditioned media were collected, centrifuged at 12,000× *g* for 10 min to remove bacteria and cellular debris, and stored at −80 °C until use. For indirect treatment of ependymal cells, the supernatant from BCG-stimulated BV2 cells (denoted as BCG+BV2-CM) or PBS-treated BV2 cells (denoted as BV2-CM and used as the control) was applied.

### 2.5. Transcriptomic Profiling and Bioinformatics Analysis

Total RNA was extracted from ependymal cells treated directly with BCG or indirectly with BCG+BV2-CM using TRIzol reagent (Invitrogen, Carlsbad, CA, USA). RNA quality was assessed using an Agilent 2100 Bioanalyzer (Agilent Technologies, Santa Clara, CA, USA; RIN > 8.0 required). Library preparation was performed using the NEBNext Ultra II RNA Library Prep Kit (New England Biolabs, Ipswich, MA, USA) and sequenced on an Illumina NovaSeq 6000 platform (Illumina, San Diego, CA, USA; 150 bp paired-end reads). Sequence reads were aligned to the mouse reference genome (GRCm38) using STAR aligner (2.7.11b). Differential expression analysis was performed using DESeq2 with thresholds of |log_2_FC| > 1 and adjusted *p*-value < 0.05. Functional enrichment analysis of KEGG pathways was conducted using clusterProfiler.

### 2.6. Integration with Single-Cell RNA Sequencing Data

Previously published single-cell RNA sequencing data from BCG-induced TBM mice (GSE199649) were reanalyzed [14]. The ependymal cell cluster was identified based on expression of canonical markers. Batch effects were corrected using Harmony integration. Differential expression analysis was performed using the MAST framework. Intersection analysis was conducted to identify common differentially expressed genes (DEGs) between in vitro treatments and in vivo single-cell data.

### 2.7. Quantitative PCR and Western Blot Analysis

Total RNA was extracted and reverse transcribed using the PrimeScript RT reagent Kit (Takara, Dalian, China). Quantitative PCR was performed using SYBR Green Master Mix (Roche, Basel, Switzerland) on a QuantStudio 6 Flex system (Applied Biosystems, Foster City, CA, USA). Gapdh was used as the housekeeping gene for normalization. Primer sequences are provided in Supplementary Table S1. For Western blotting, proteins were separated by SDS-PAGE, transferred to PVDF membranes (Millipore, Billerica, MA, USA), and probed with primary antibodies against Hmgcs2 (1:1000; Abcam), acetylated α-tubulin (1:2000; Abcam), and Gapdh (1:5000; Proteintech, Wuhan, China), followed by incubation with appropriate HRP-conjugated secondary antibodies (Proteintech).

### 2.8. Functional Assessments

Cell apoptosis was detected using an Annexin V-FITC/PI Apoptosis Detection Kit (BD Biosciences, San Jose, CA, USA) according to the manufacturer’s instructions. Briefly, cells were resuspended in 1× binding buffer and incubated with Annexin V-FITC and propidium iodide (PI) for 15 min at room temperature in the dark. Samples were analyzed immediately using a BD FACS Celesta flow cytometer (BD Biosciences, San Jose, CA, USA). Data were processed with FlowJo software (version 10.8.1; Tree Star, Ashland, OR, USA), and apoptotic rates were defined as the percentage of Annexin V-positive cells (including early and late apoptotic populations).

To evaluate the protective effect of sodium butyrate (NaB; Sigma-Aldrich), ependymal cells were pretreated with 1 mM NaB for 24 h. After pretreatment, cells were exposed either directly to BCG (MOI = 10) or indirectly to conditioned medium from BCG-stimulated BV2 cells (BCG+BV2-CM) for an additional 24 h. Control groups received equivalent volumes of PBS or unconditioned BV2 medium.

### 2.9. Statistical Analysis

Data are presented as mean ± SEM from at least three independent experiments. Statistical analyses were performed using GraphPad Prism 8.0 (GraphPad Software, San Diego, CA, USA). Normality was assessed using the Shapiro–Wilk test. Comparisons between two groups were analyzed using Student’s *t*-test (parametric) or Mann–Whitney U test (non-parametric). Multiple group comparisons were performed using one-way ANOVA followed by Tukey’s post hoc test. A *p*-value < 0.05 was considered statistically significant.

## 3. Results

### 3.1. Successful Construction of a TBM Mouse Model via Tail Vein Injection of BCG

**Figure 1:**
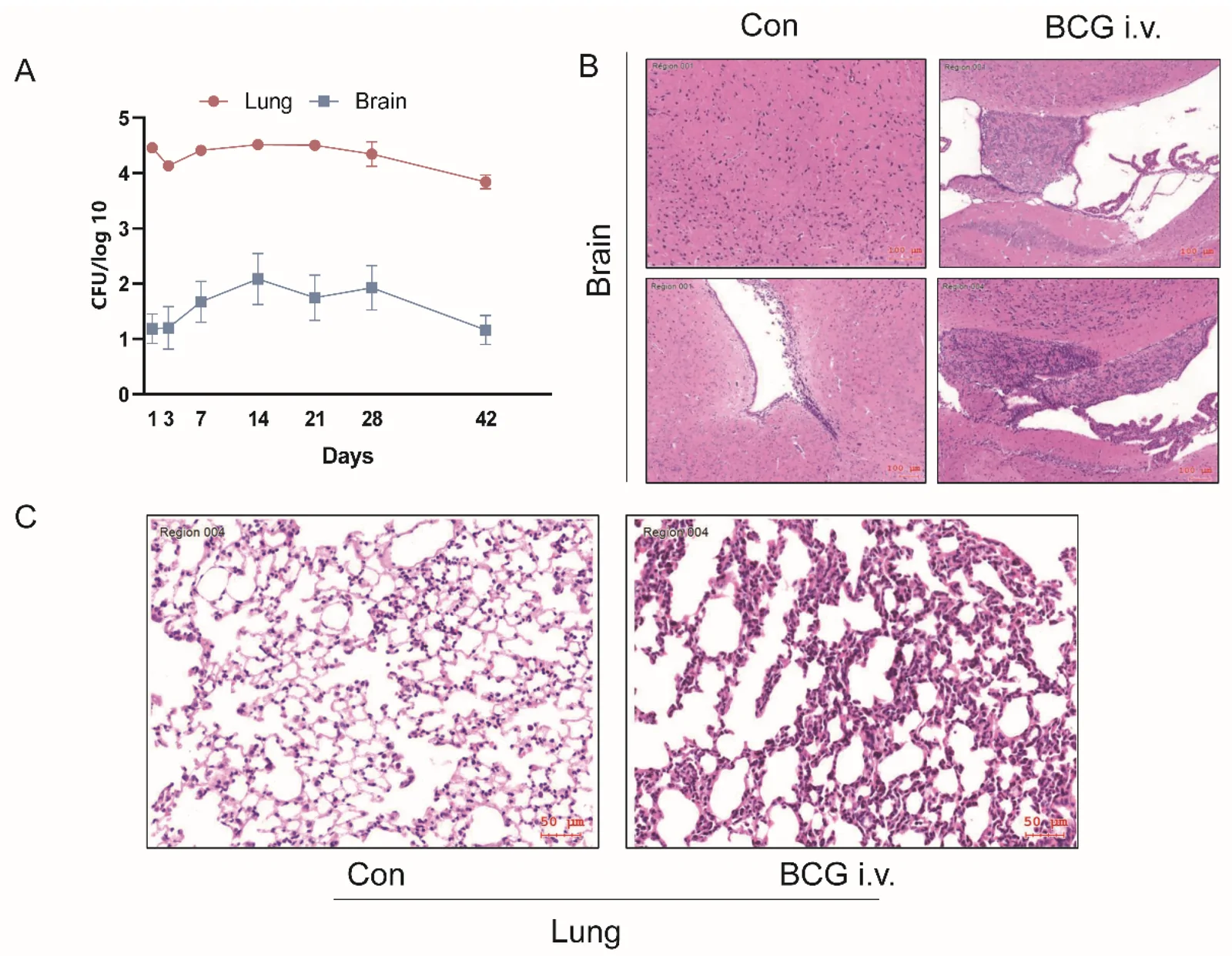
Construction and validation of the TBM mouse model. (**A**) BCG injection into the tail vein of mice resulted in the sustained presence of *Mycobacterium bovis* in both brain and lung tissues, indicating successful systemic infection; (**B**) hematoxylin and eosin (H&E) staining of brain sections revealed marked inflammatory cell infiltration surrounding the ventricles, indicative of CNS involvement; (**C**) H&E staining of lung sections showed severe inflammation with disrupted alveolar structures and unclear alveolar boundaries, confirming pulmonary infection and inflammation. Mice were used in this study; *n* = 6 mice per group (3 BCG-infected + 3 control for histopathology).

To establish a tuberculous meningitis (TBM) mouse model, BCG was administered via tail vein injection. The results confirmed the sustained presence of *Mycobacterium bovis* in both brain and lung tissues, indicating successful systemic infection (Figure 1A). Hematoxylin and eosin (H&E) staining of brain sections showed significant inflammatory cell infiltration around the ventricles, suggesting central nervous system involvement (Figure 1B). H&E staining of lung sections revealed severe inflammatory responses, disrupted alveolar structures, and blurred alveolar boundaries, confirming pulmonary infection and inflammation (Figure 1C). These results demonstrate the successful establishment of a TBM mouse model with key pathological features.

### 3.2. Isolation, Culture, and Characterization of Primary Ependymal Cells

**Figure 2:**
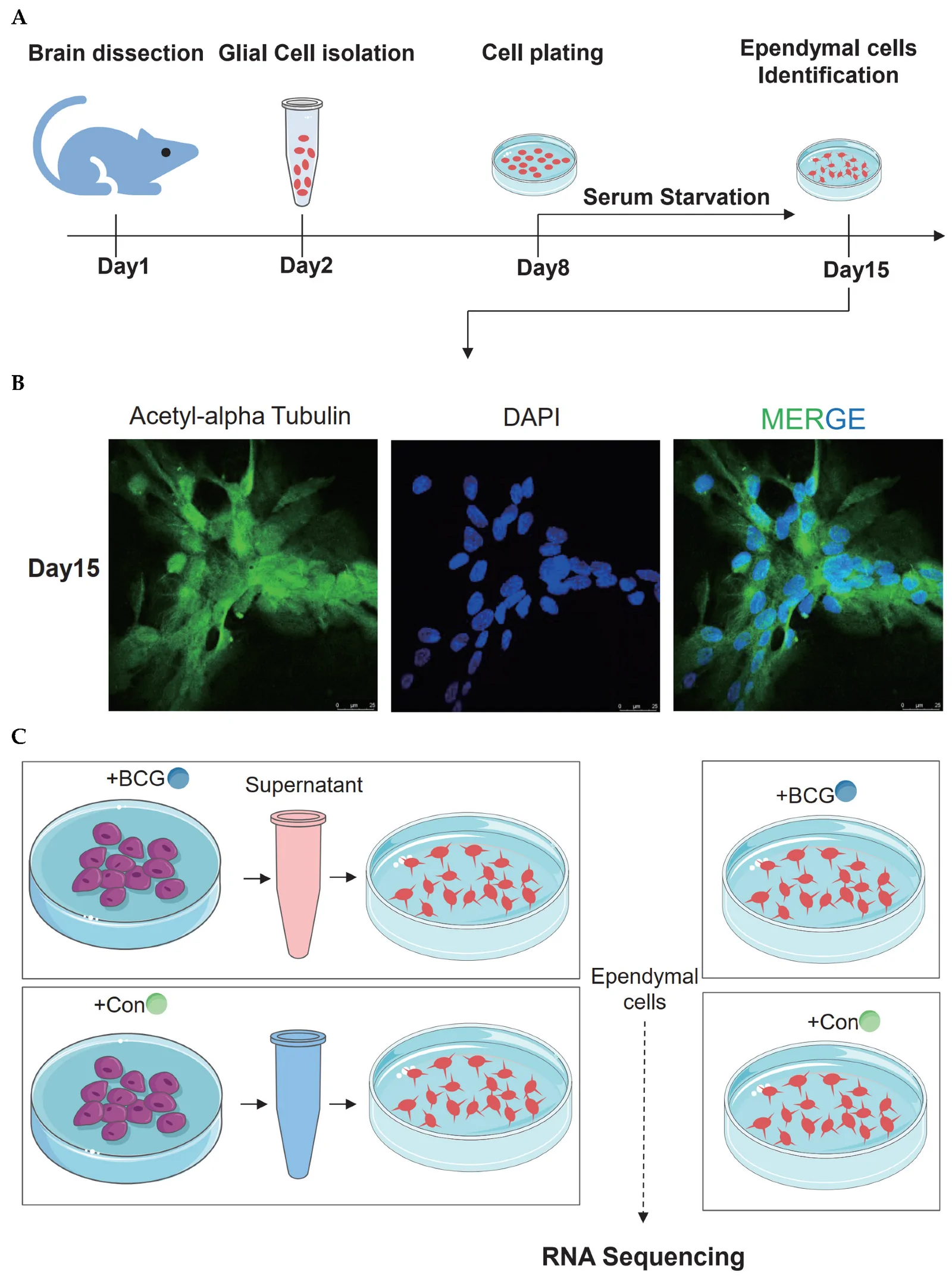
Isolation, culture, and treatment of ependymal cells. (**A**) Schematic diagram of the isolation and primary culture process of ependymal cells from the brain ventricles of mice; (**B**) immunofluorescence staining of ependymal cells on day 15, showing typical cilia structures and cell morphology; (**C**) experimental design: UP group treated ependymal cells with supernatant from BCG-stimulated BV2 microglia; DOWN group treated ependymal cells with supernatant from PBS-treated BV2, to distinguish the direct and indirect effects of BCG.

To investigate the mechanisms of BCG infection at the cellular level, we established a primary culture system for mouse ependymal cells. Figure 2A illustrates the schematic procedure for isolating and culturing ependymal cells from mouse brain ventricles. After 15 days of culture, immunofluorescence staining confirmed the successful cultivation of ependymal cells, showing typical cilia structures and morphological characteristics (Figure 2B). To distinguish between the direct and indirect effects of BCG on ependymal cells, we designed two treatment groups: the UP group received supernatant from BCG-stimulated BV2 microglia, while the DOWN group received supernatant from PBS-treated BV2 cells (Figure 2C). This experimental setup allowed us to specifically investigate the microglia-mediated indirect effects of BCG on ependymal cells.

### 3.3. Microglia Significantly Amplify BCG-Induced Transcriptional Changes in Ependymal Cells

**Figure 3:**
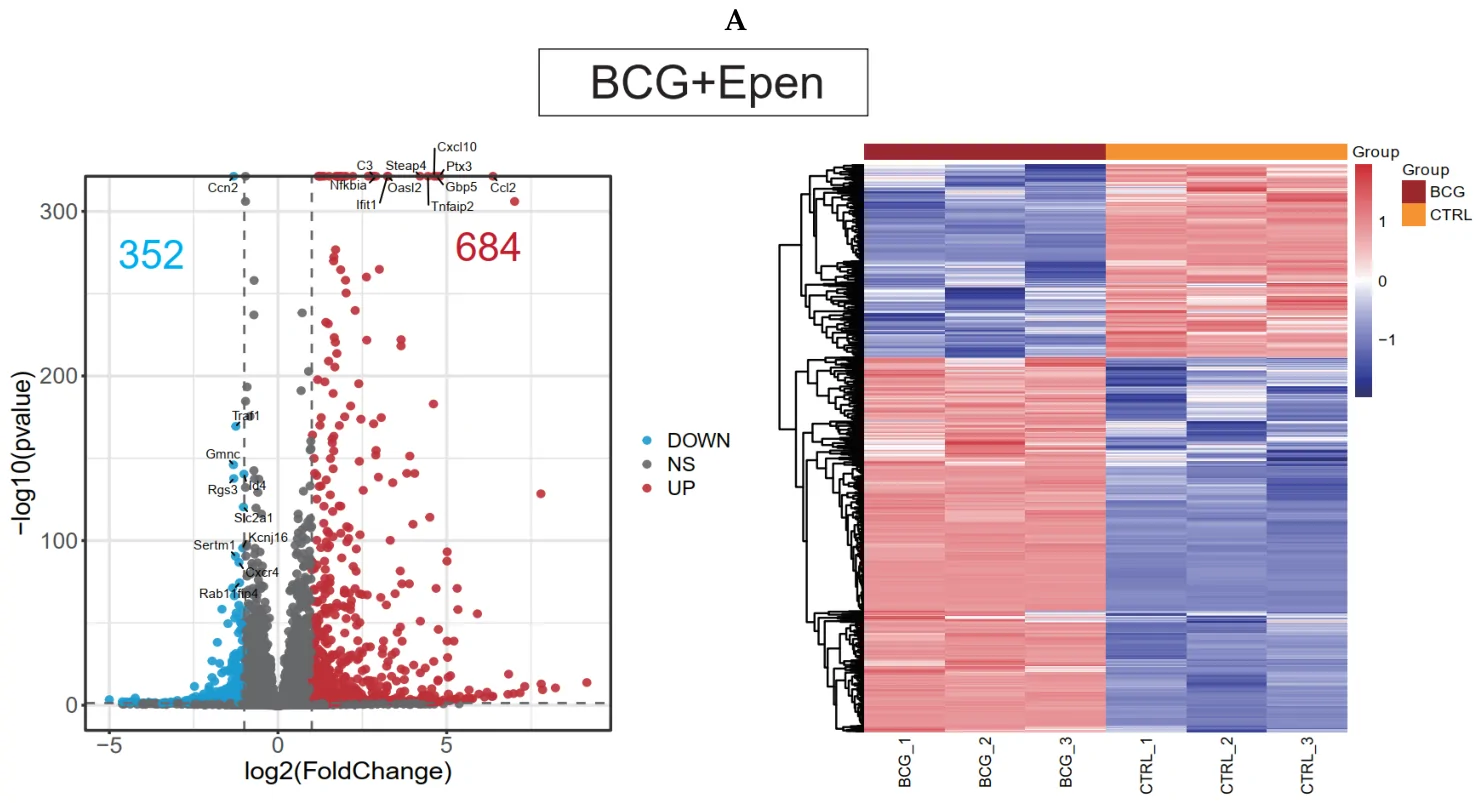

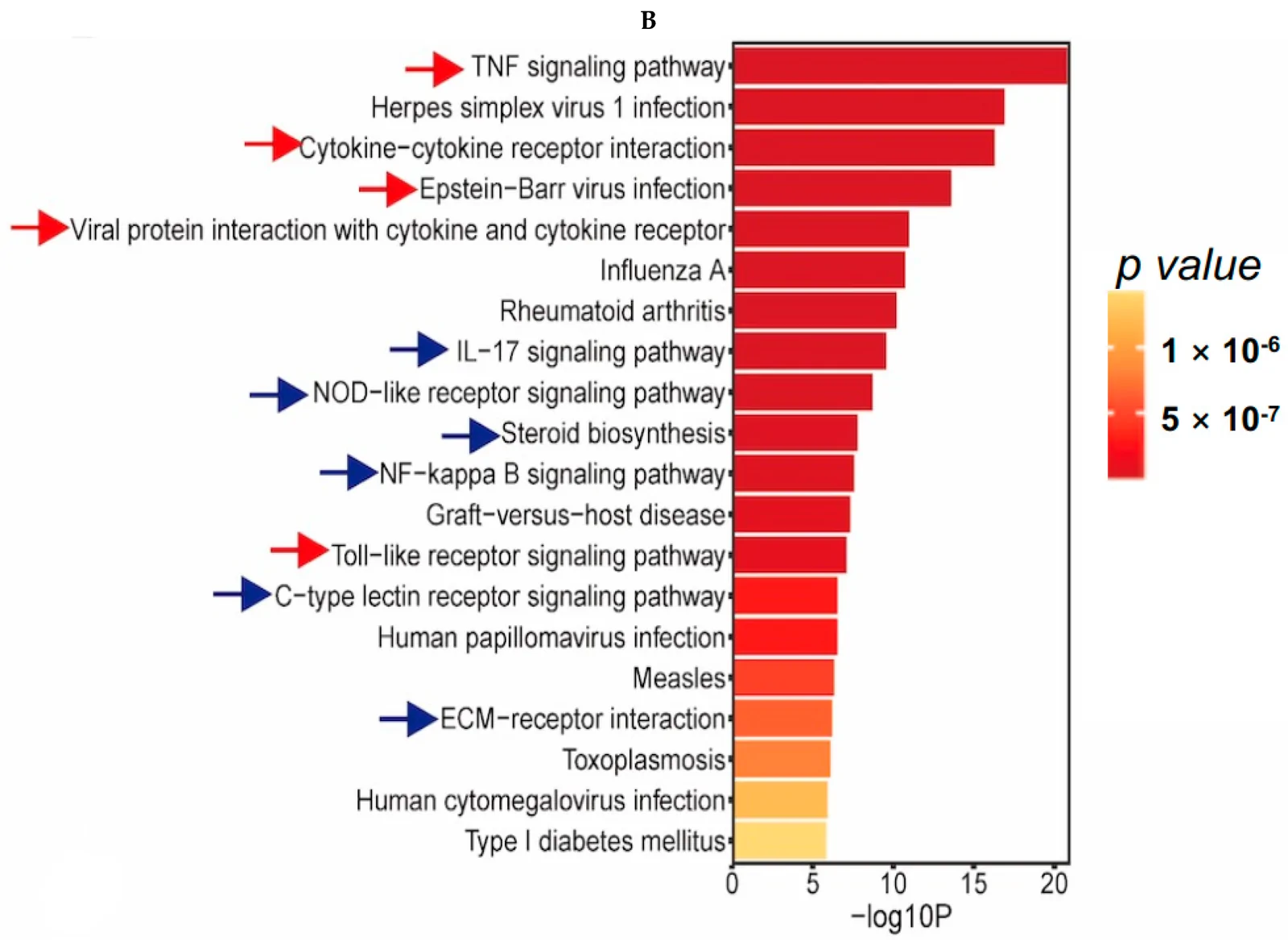

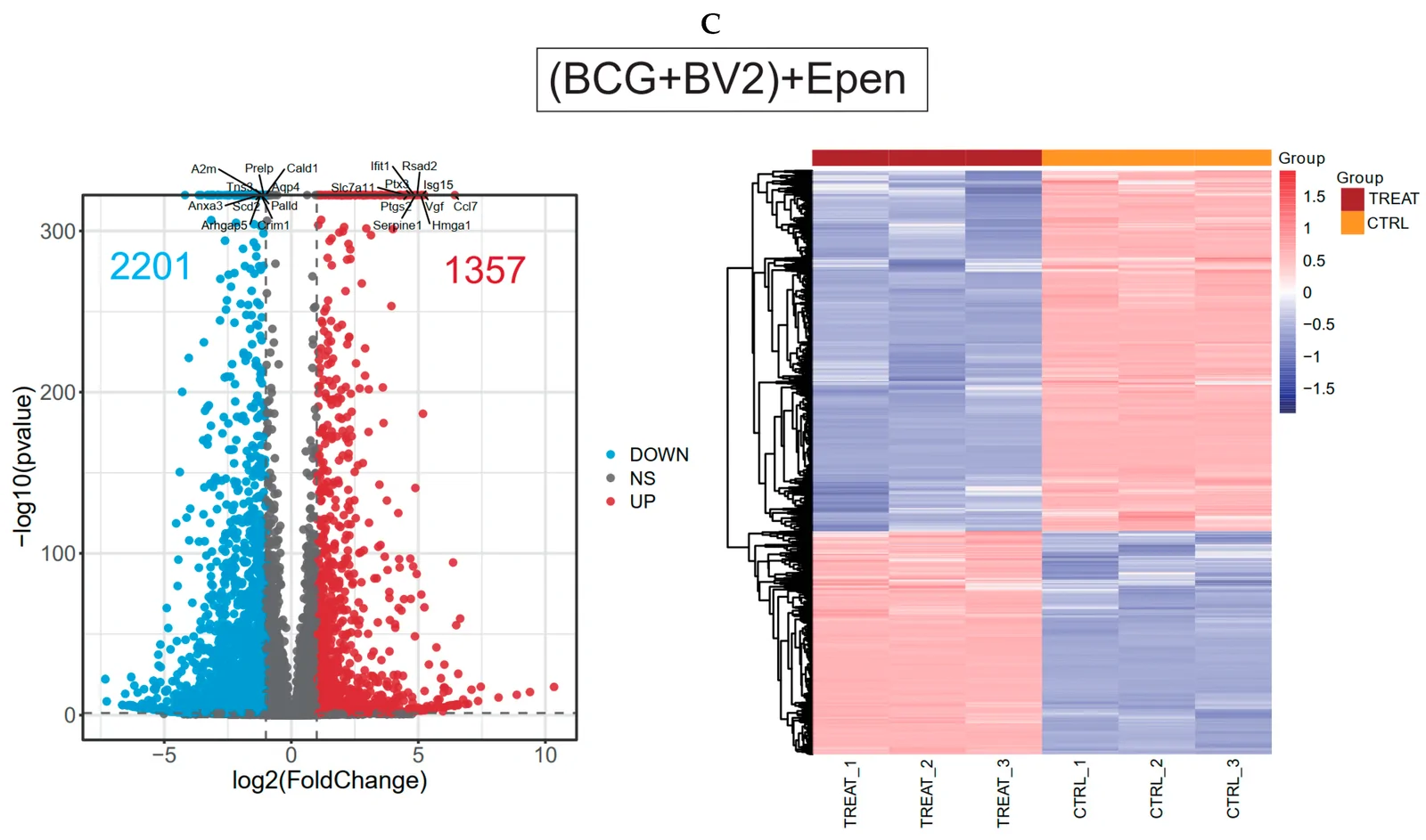

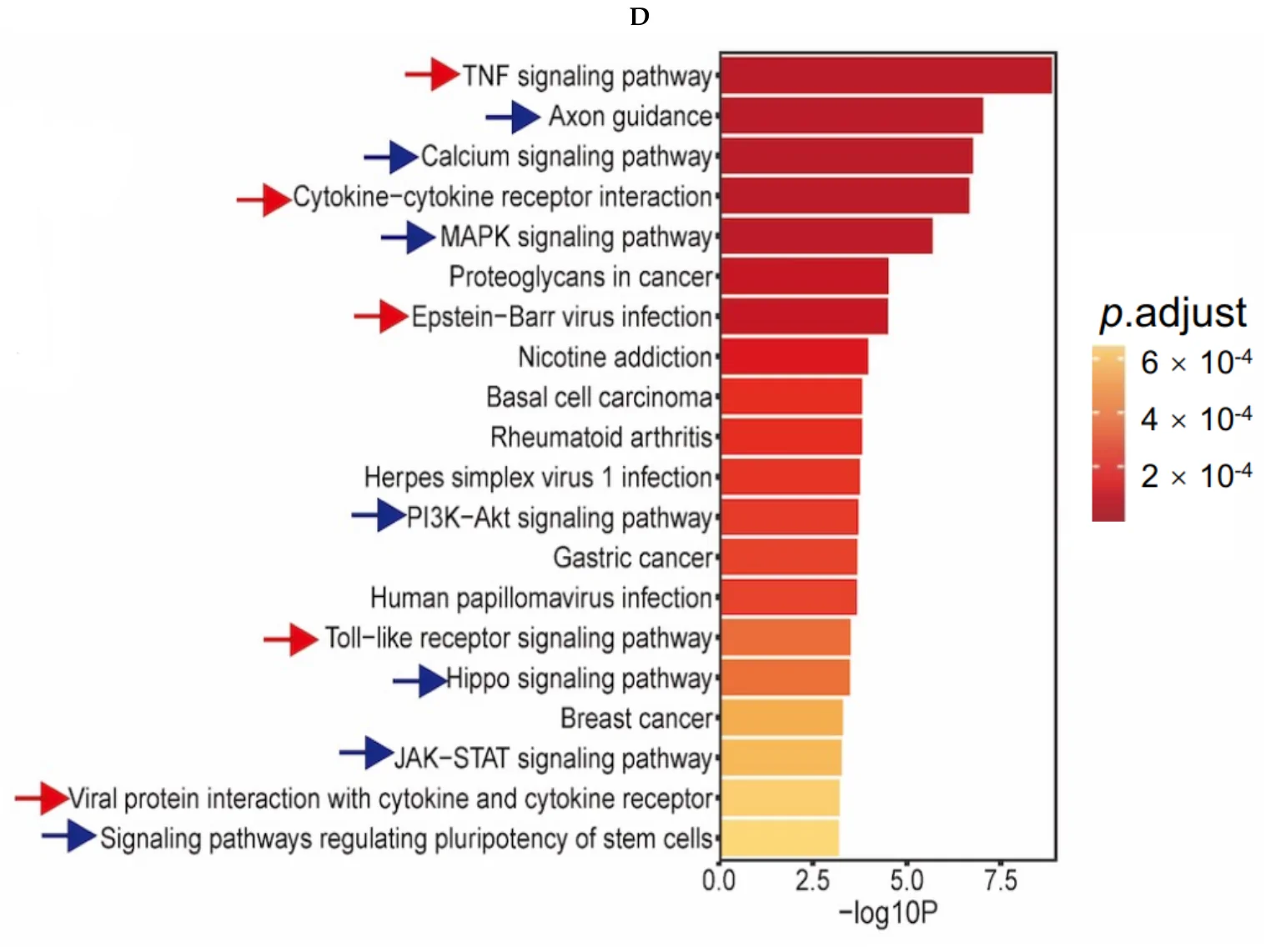
Analysis of differentially expressed genes and pathway enrichment in ependymal cells treated with BCG directly and indirectly via BV2. (**A**) The left panel of this figure presents a volcano plot depicting the differential gene expression in ependymal cells treated directly with BCG. The plot highlights 352 upregulated genes and 684 downregulated genes, indicating significant transcriptional changes upon direct BCG exposure. The right panel displays a heatmap of hierarchical clustering for these differentially expressed genes, revealing distinct expression patterns among various genes. (**B**) This panel illustrates the KEGG pathway enrichment analysis results for the significantly differentially expressed genes in ependymal cells treated directly with BCG. The pathways are ranked by their adjusted *p*-value, with red arrows indicating common pathways enriched in both treatment groups, such as the TNF signaling pathway. Blue arrows point to pathways specifically enriched in the BCG direct treatment group, such as Herpes simplex virus 1 infection, suggesting specific impacts of BCG on ependymal cells. (**C**) The left panel of this figure shows a volcano plot of differential gene expression in ependymal cells treated indirectly with BCG via BV2 activation. It identifies 2201 upregulated genes and 1357 downregulated genes, indicating substantial transcriptional alterations due to indirect BCG exposure through BV2. The right panel provides a heatmap of hierarchical clustering for these differentially expressed genes, highlighting different expression profiles. **(D**) This panel presents the KEGG pathway enrichment analysis for the significantly differentially expressed genes in ependymal cells treated indirectly with BCG via BV2. Like panel B, pathways are ranked by their adjusted *p*-value. Red arrows denote common pathways enriched in both treatment groups, such as the TNF signaling pathway, while blue arrows indicate pathways specifically enriched in the BCG+BV2 treatment group, like cytokine-cytokine receptor interaction. These findings suggest that BV2-mediated indirect effects of BCG on ependymal cells may trigger distinct biological responses compared to direct BCG treatment (*n* = 3 independent biological replicates per condition for bulk RNA-seq).

Using the established ependymal cell culture system, we investigated the role of microglia in BCG-induced transcriptional regulation. Cells were treated either directly with BCG or indirectly with conditioned medium from BCG-stimulated BV2 microglia (BCG+BV2-CM), followed by transcriptome sequencing. Direct BCG treatment induced 1036 differentially expressed genes (DEGs) in ependymal cells, including 352 upregulated and 684 downregulated genes (Figure 3A). Notably, indirect treatment with BCG+BV2-CM induced 3558 DEGs, comprising 1357 upregulated and 2201 downregulated genes, representing a 3.4-fold increase in transcriptional alterations compared to direct treatment (Figure 3C). KEGG pathway enrichment analysis revealed that pathways like TNF signaling pathway, Toll-like receptor, and cytokine–cytokine receptor interaction were significantly enriched in both treatment groups, while each treatment also showed specific enriched pathways (Figure 3B,D). For BCG + Ependymal groups, specific pathways like IL-17 signaling, NOD-like receptor, and NF-kappa B singling were enriched. On the other side, pathways like Axon guidance, MAPK, PI3K-Akt, and Hippo signaling were enriched in BCG+BV2+Ependymal cells. These findings suggest that microglia significantly amplify the transcriptional regulatory effects of BCG on ependymal cells through secreted factors.

**Figure 4:**
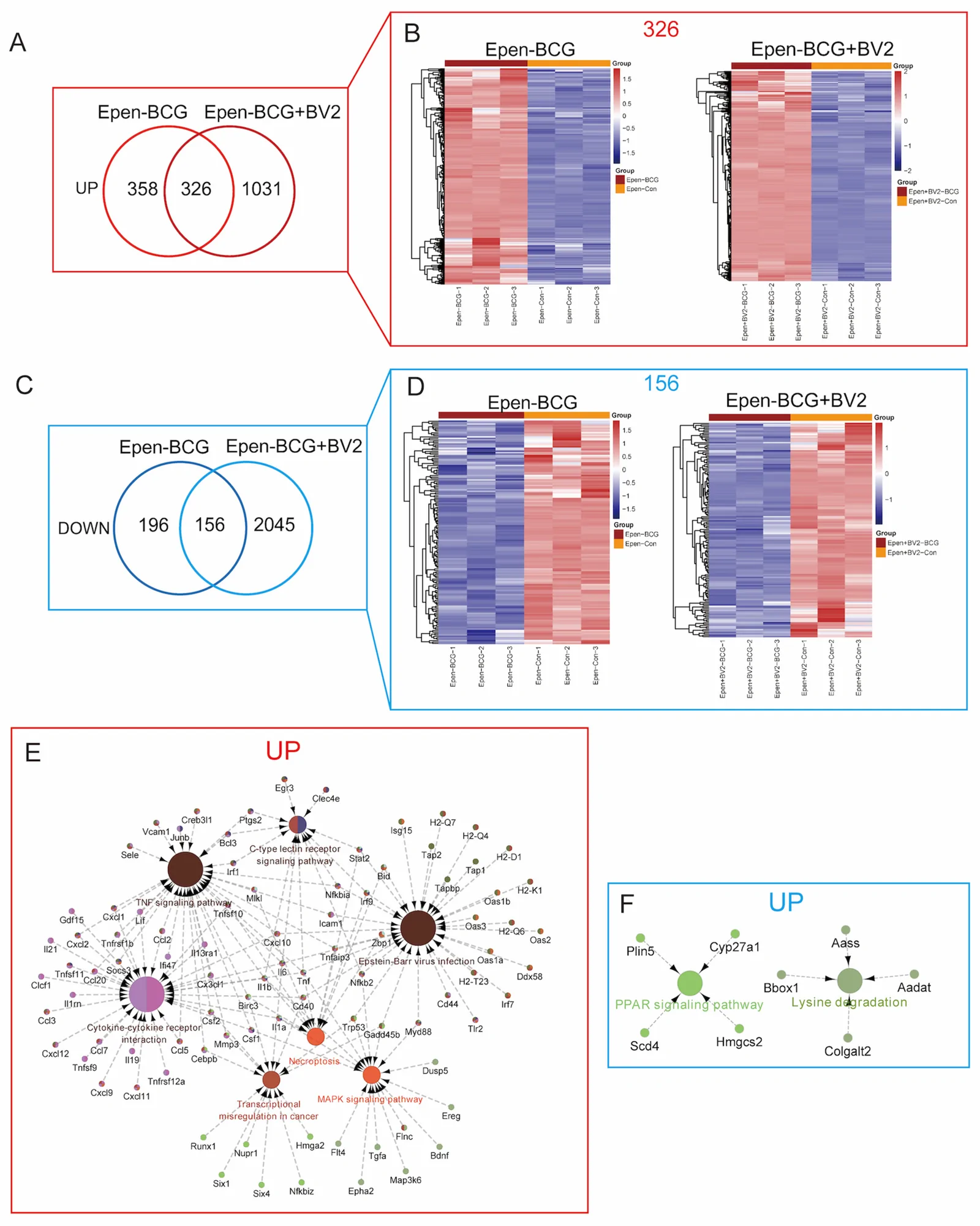
Identification and functional analysis of common differentially expressed genes in ependymal cells treated with BCG directly and indirectly via BV2. (**A**) Venn diagram illustrating the intersection of upregulated genes between ependymal cells treated with BCG directly (Epen-BCG) and those treated with BCG indirectly via BV2 (Epen-BCG+BV2). A total of 326 genes were found to be commonly upregulated in both treatment groups. (**B**) Heatmap representing the expression levels of the 326 commonly upregulated genes identified in panel A. The heatmap provides a visual representation of the gene expression patterns across the two treatment conditions, with color gradients indicating the level of expression changes. (**C**) Venn diagram showing the intersection of downregulated genes between the Epen-BCG and Epen-BCG+BV2 groups. This analysis revealed 156 genes that were commonly downregulated. (**D**) Heatmap depicting the expression levels of the 156 commonly downregulated genes identified in panel C. Similar to panel B, this heatmap visualizes the expression patterns of these genes under the two different treatment conditions. (**E**) Gene-pathway network diagram for the upregulated genes. This network map illustrates the interconnected relationships among the 326 upregulated genes, highlighting their potential roles in various biological pathways and processes. Notably enriched pathways include the MAPK signaling pathway, which involves genes such as Eg3, Cdk4, and Cdk6, indicating their involvement in cell signaling and response to extracellular stimuli. Additionally, the Th2-type immune response pathway is represented, with genes like Gata3 and Stat6 playing crucial roles in immune cell activation and differentiation. (**F**) Gene-pathway network diagram for the downregulated genes. This network map provides insights into the interconnected pathways and functional associations of the 156 downregulated genes, which may be implicated in the cellular responses to the treatments. Enriched pathways include the PPAR signaling pathway, with genes such as Plnp5, Hmgcs2, and Aass potentially affecting cellular adhesion and migration. The Lysine degradation pathway is also highlighted, with genes like Bbox1 and Colgalt2 possibly contributing to the breakdown and recycling of amino acids within the cell.

Further analysis identified 326 commonly upregulated and 156 commonly downregulated genes between the two treatment conditions (Figure 4A,C). Heatmaps visualized the expression patterns of these common DEGs (Figure 4B,D). Gene-pathway network analysis revealed that the upregulated genes were primarily involved in the MAPK signaling pathway (e.g., Eg3, Cdk4, Cdk6) and Th2-type immune response (e.g., Gata3, Stat6) (Figure 4E). The downregulated genes were mainly enriched in the PPAR signaling pathway (e.g., Plnp5, Hmgcs2, Aass) and lysine degradation pathway (e.g., Bbox1, Colgalt2) (Figure 4F). Together, these results demonstrate that microglia-derived soluble factors profoundly exacerbate BCG-induced transcriptional dysregulation in ependymal cells, amplifying immune and inflammatory pathways while suppressing key metabolic processes.

### 3.4. Integrated Single-Cell Transcriptome Analysis Identifies Core DEGs in Ependymal Cells in TBM

**Figure 5:**
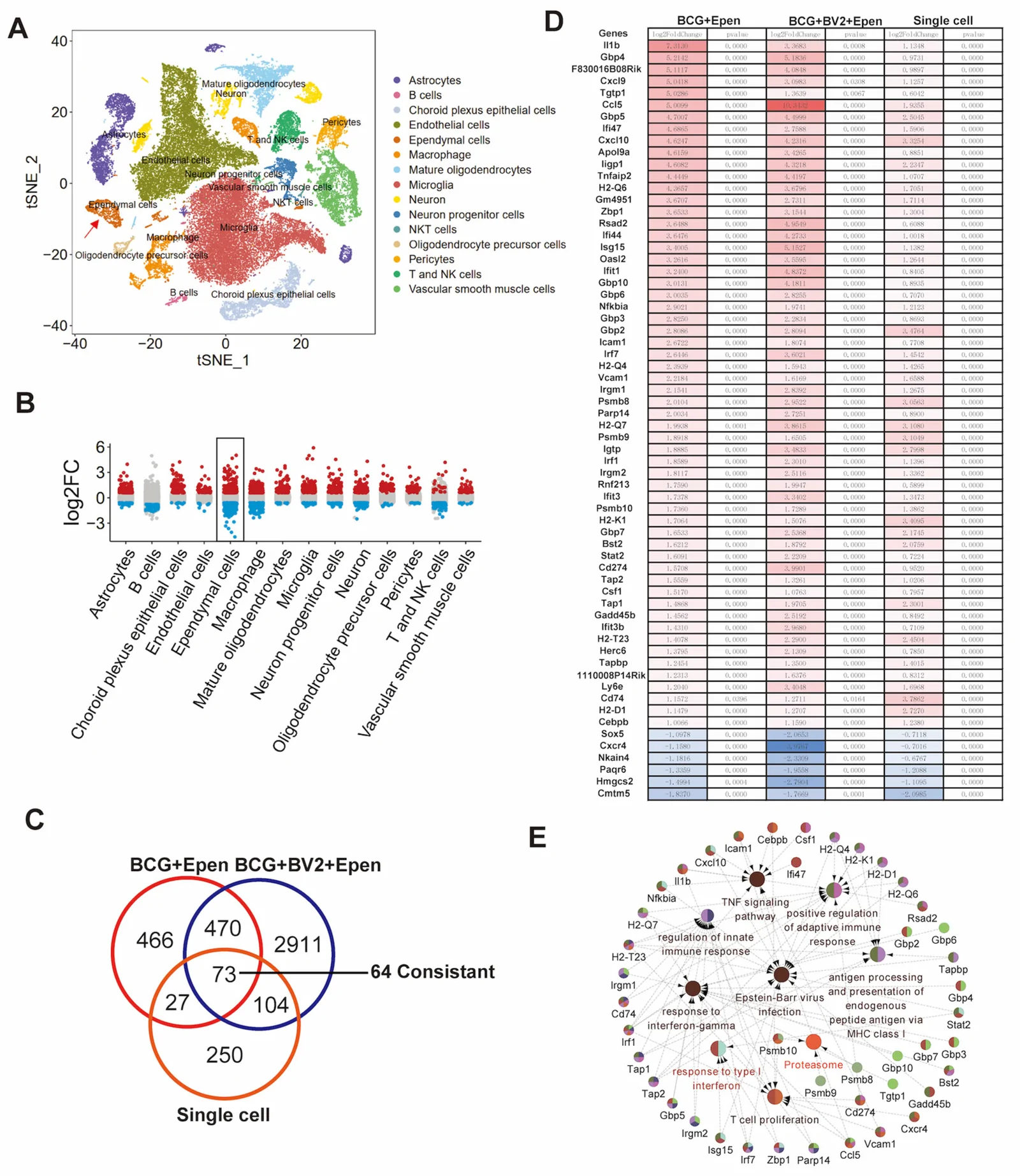
Integration of single-cell data to identify core differentially expressed genes. (**A**) T-SNE plot showing the classification of various cell types identified from single-cell RNA sequencing data. The plot distinguishes between different cell populations including astrocytes, oligodendrocytes, ependymal cells, microglia, and others, each represented by distinct clusters (mice were used in this study *n* = 3 mice per group from the GSE199649 dataset (3 BCG + 3 control)). (**B**) t-SNE plot specifically highlighting ependymal cells among the identified cell types, with a clear demarcation of their expression profiles. The plot indicates that ependymal cells exhibit the highest number of differentially expressed genes compared to other cell types. (**C**) Venn diagram illustrating the intersection of differentially expressed genes between the two treatment groups (BCG+Epen and BCG+BV2+Epen) and the single-cell sequencing data. A total of 73 common differential genes were identified, with 64 genes showing a consistent trend of expression change across all three datasets. (**D**) Heatmap displaying the fold change and *p*-values for differentially expressed genes across three datasets: BCG+Epen, BCG+BV2+Epen, and single-cell RNA sequencing data. The heatmap shows the expression trends of these genes in the three datasets. (**E**) Regulatory network diagram depicting the interactions between differentially expressed genes and their associated pathways. Notably enriched pathways include the TNF signaling pathway involving genes such as Tnfα and Il6, which are linked to the regulation of adaptive immune responses. Additionally, the Jak-STAT signaling pathway is highlighted, with genes like Stat3 and Stat6 playing roles in cytokine signaling and immune cell differentiation. The diagram also indicates the involvement of genes such as Cxcl10 and Ccl21 in chemokine signaling, and H2-Q4 and H2-Q7 in antigen processing and presentation, suggesting their potential roles in immune regulation and cell signaling in response to BCG treatments.

To further evaluate the in vivo relevance of the in vitro findings, we integrated the in vitro differentially expressed genes with the published single-cell sequencing data of BCG-infected mouse brain tissues (GSE199649). T-SNE clustering successfully distinguished major CNS cell types such as astrocytes, oligodendrocytes, ependymal cells, and microglia (Figure 5A), with ependymal cells showing the most significant transcriptional changes among all cell types (Figure 5B). Intersection analysis identified 73 common differentially expressed genes between in vitro treatment groups and single-cell data, and 64 of these genes showed consistent expression trends in the three datasets (Figure 5C). A heatmap shows the expression trends of these genes in the three datasets (Figure 5D). It should be noted that log_2_FC values cannot be directly compared numerically across datasets due to the essential differences in standardization methods and statistical framework between bulk RNA-seq and single-cell MAST analysis. Therefore, the heatmap was designed to demonstrate the directional consistency of gene expression changes rather than to make quantitative comparisons across platforms. It should be noted that the original study of GSE199649 did not report the specific cell number of the ependymal cell population, and ependymal cells usually account for a very low proportion (<1%) in single-cell datasets, which may affect the statistical power of differential expression analysis. Therefore, the results of this pooled analysis should be viewed as supporting circumstantial evidence of the in vitro findings and not as independent confirmation. Nonetheless, genes that were consistently dysregulated both in vitro and in vivo were significantly enriched in immune-related pathways such as TNF signaling, JAK-STAT signaling, chemokine signaling, and antigen processing and presentation (Figure 5E), findings that were consistent with our in vitro transcriptome analysis (Response to Reviewer 3, Comment 6).

### 3.5. Sodium Butyrate Attenuates BCG-Induced Ependymal Cell Dysfunction by Restoring Hmgcs2 Expression

**Figure 6:**
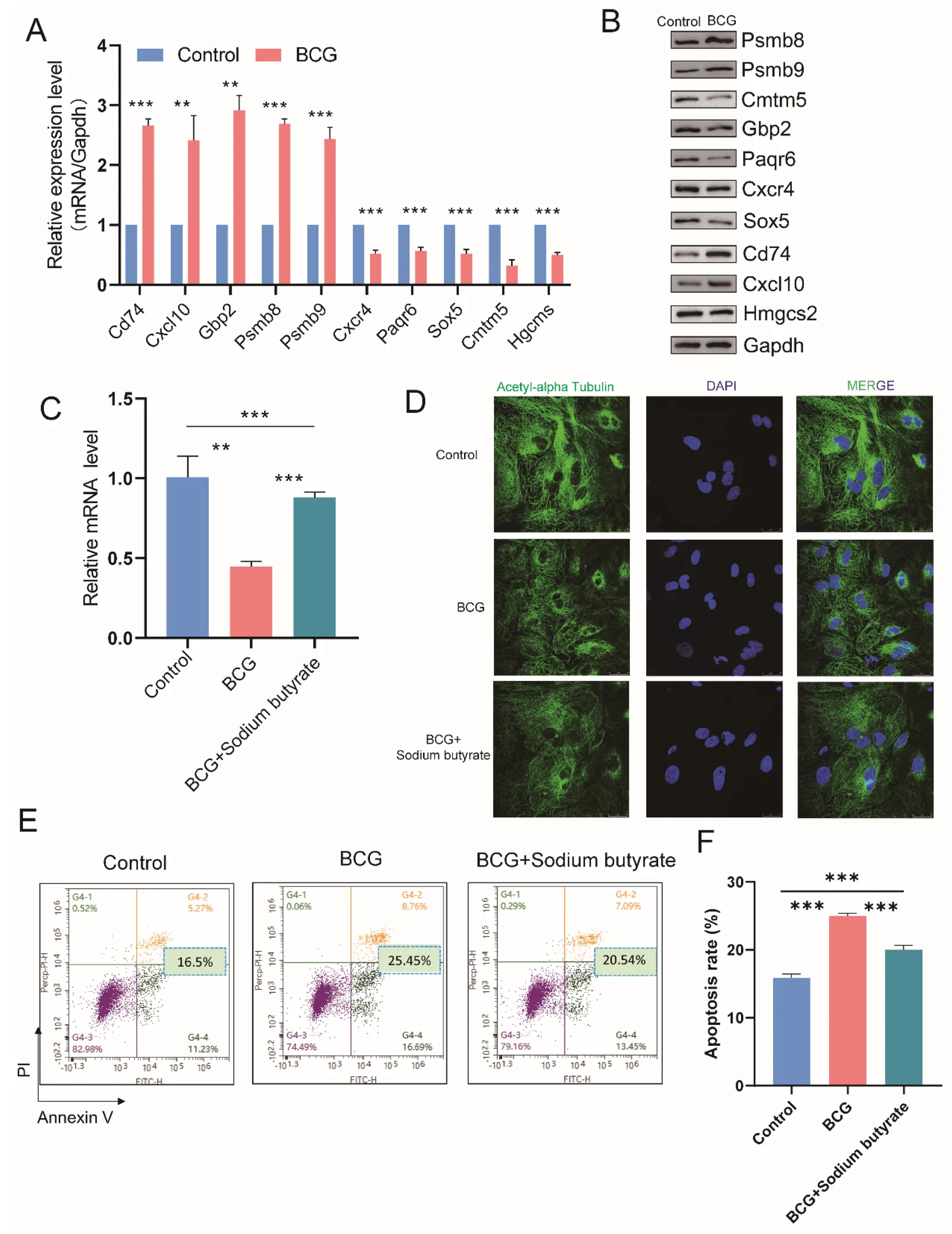
Sodium butyrate improves ependymal cell dysfunction by activating Hmgcs2. (**A**) Bar graph showing the relative mRNA expression levels of five upregulated genes (Cd74, Gbp2, Psmb9, Psmb8, and Cxcl10) and five downregulated genes (Cxcr4, Sox5, Hmgcs2, Paqr6, and Cmtm5) in ependymal cells treated with BCG compared to control cells. The expression levels were determined by qPCR, normalized to the housekeeping gene Gapdh, and presented as mean ± SEM. Statistical significance is indicated by asterisks. (**B**) Western blot analysis confirming the protein expression changes in the same ten genes in response to BCG treatment. The blots are probed with antibodies against the target proteins and normalized to the expression of the housekeeping protein Gapdh. (**C**) Bar graph depicting the relative mRNA level of the downregulated gene Hmgcs2 in control, BCG-treated, and BCG-treated with sodium butyrate ependymal cells. The results demonstrate that sodium butyrate can significantly restore the expression of Hmgcs2, which is reduced by BCG treatment. (**D**) Immunofluorescence images showing the cilia length in ependymal cells under different treatment conditions. The images compare the cilia length in control, BCG-treated, and BCG-treated with sodium butyrate cells, stained with acetyl-alpha tubulin (green) and DAPI (blue) for nuclei. The treatment with BCG shortens the cilia, while sodium butyrate partially restores the cilia length. Scale bar: 25 μm. (**E**) Flow cytometry analysis of cell apoptosis in ependymal cells treated with BCG, BCG plus sodium butyrate, and under control conditions. The plots show the distribution of cells stained with Annexin V (green) and propidium iodide (PI, red), which are used to distinguish live cells (Annexin V−/PI−), early apoptotic cells (Annexin V+/PI−), late apoptotic cells (Annexin V+/PI+), and necrotic cells (Annexin V−/PI+). The results demonstrate the effect of BCG and BCG plus sodium butyrate on inducing apoptosis in ependymal cells. (**F**) Bar graph summarizing the apoptotic rate of ependymal cells under different treatment conditions, as determined by flow cytometry. The results show a significant increase in apoptosis induced by BCG treatment, which is significantly reduced when sodium butyrate is added, indicating a protective effect of sodium butyrate against BCG-induced cell death (** *p* < 0.01, *** *p* < 0.001). Data are presented as mean ± SEM from three independent experiments (*n* = 3 per group).

Given the central role of metabolic suppression in ependymal injury, we focused on Hmgcs2—a downregulated enzyme in ketogenesis—as a potential mediator of dysfunction. Verification by qPCR and Western blot confirmed that BCG+BV2-CM treatment significantly downregulated Hmgcs2 expression at both mRNA and protein levels (Figure 6A,B). Functional analysis demonstrated that BCG challenge induced substantial shortening of the ependymal cell cilia (Figure 6D) and significantly increased the apoptotic rate (Figure 6E,F). We next asked whether these defects could be reversed through the therapeutic modulation of Hmgcs2. Notably, sodium butyrate (NaB) pretreatment effectively restored Hmgcs2 expression (Figure 6C). At the functional level, NaB treatment markedly prevented BCG-induced ciliary shortening (Figure 6D) and substantially reduced the apoptosis rate (Figure 6E,F). These results indicate that Hmgcs2 downregulation is mechanistically linked to BCG-induced ependymal cell dysfunction, and that sodium butyrate confers structural and functional protection by upregulating Hmgcs2 expression.

## 4. Discussion

Tuberculous meningitis (TBM) remains one of the most devastating forms of tuberculosis, characterized by high mortality and severe neurological sequelae even among survivors [27]. A critical yet often underappreciated feature of TBM is the disruption of cerebrospinal fluid (CSF) homeostasis, leading to cerebral edema, elevated intracranial pressure, and hydrocephalus—complications that significantly contribute to poor outcomes [28,29]. The pathological basis of these processes involves not only direct bacterial invasion but also dysregulated host immune responses that exacerbate neural tissue damage [1,2].

Microglia are the core resident immune effector cells of the central nervous system (CNS) [30]. In tuberculous meningitis (TBM), following the hematogenous dissemination of Mycobacterium tuberculosis across the blood–brain barrier, microglia are among the first CNS cells to recognize and internalize the pathogen [31]. However, mycobacteria are not only phagocytosed by microglia but can also survive and replicate intracellularly, rendering microglia a potential pathogen reservoir within the CNS [32]. Infection triggers robust microglial activation, characterized by massive release of pro-inflammatory cytokines (TNF-α, IL-1β, IL-6) and IL-1β maturation via NLRP3 inflammasome activation [33]. Furthermore, mycobacterial infection can induce microglial polarization toward the pro-inflammatory M1 phenotype, a process partially mediated through macrophage–microglia interactions [34]. Functionally, microglial activation in TBM exhibits a “double-edged sword” effect: appropriate activation helps restrict pathogen dissemination, yet much of the tissue damage in TBM arises from microglia-driven “non-productive” neuroinflammation—a state in which robust inflammatory activation fails to clear the pathogen effectively, instead causing excessive tissue injury [35]. Persistently activated microglia overproduce TNF-α and IL-1β, damaging adjacent neurons, disrupting the blood–brain barrier, promoting leukocyte infiltration, and inducing oxidative stress [36]. Recent single-cell transcriptomic studies have further revealed that the cerebrospinal fluid of TBM patients is enriched with highly inflammatory microglia-like macrophages, with complement-activated subsets closely associated with persistent neuroinflammation [37]. Nevertheless, how microglial activation specifically affects ependymal cells—the key barrier cells at the brain–CSF interface—remains poorly understood, constituting the central entry point of the present study.

Neuroinflammation plays a central role in TBM pathogenesis, with microglial activation being a key driver of disease progression. Activated microglia release pro-inflammatory cytokines such as TNF-α and IL-1β, which can induce neuronal apoptosis and contribute to cognitive impairment [3]. Moreover, microglia-mediated inflammation disrupts the blood–brain barrier integrity and promotes vasogenic edema, further compounding neurological dysfunction [4]. Therefore, targeting neuroinflammatory mechanisms has emerged as a promising adjunctive strategy in TBM management.

Recent advances have highlighted the importance of ependymal cells in maintaining CSF dynamics and barrier functions. These cells, lining the ventricular system, facilitate CSF flow through ciliary beating, regulate substance exchange, and support neurogenesis [7,8]. Our previous single-cell RNA sequencing study in a BCG-induced murine model of TBM revealed that ependymal cells undergo the most profound transcriptional alterations among all CNS cell types, suggesting their pivotal role in TBM pathophysiology [14]. In the present study, we extended these findings by establishing a TBM model via tail vein injection of BCG in mice and conducting in vitro assays with primary ependymal cells. Using transcriptomic profiling, we compared the effects of direct BCG stimulation versus microglia-conditioned BCG exposure on ependymal cells.

Notably, our results demonstrate that BCG can activate ependymal cells through both direct and microglia-mediated mechanisms. Direct BCG exposure enriched pathways such as TNF signaling, cytokine–cytokine receptor interaction, and Toll-like receptor signaling [18]. Similarly, BCG preconditioned with BV2 microglia also activated these pathways but markedly amplified the transcriptional response, increasing the number of differentially expressed genes from 1036 to 3558. This amplification effect underscores the critical role of microglia in exacerbating ependymal cell injury during TBM. Furthermore, functional pathway analysis revealed that direct BCG stimulation primarily affected IL-17 signaling, NOD-like receptor signaling, and NF-κB signaling, whereas microglia-mediated BCG exposure additionally enriched Axon guidance, MAPK, PI3K-Akt, and Hippo signaling pathways. This is despite the fact that the experiments used an immortalized BV2 microglia cell line rather than primary microglia [38]. BV2 cells are known to exhibit a persistently activated hyperinflammatory phenotype that differs from primary microglia in terms of cytokine secretion and transcriptional responses [39]. However, our parallel experiments with direct stimulation of primary ependymal cells with BCG in the absence of microglia independently confirmed Hmgcs2 downregulation, suggesting that this central finding is not BV2-dependent [40]. Future studies using primary microglia-conditioned media or microglia–ependymal co-culture systems are needed to validate these findings under more physiological conditions. These findings support the hypothesis that neuroinflammatory signaling significantly contributes to ependymal dysfunction and CSF imbalance in TBM.

Another key discovery was the identification of a common set of genes dysregulated across both in vitro and in vivo TBM models, particularly involving the ketogenesis pathway. Among these, Hmgcs2—encoding the rate-limiting enzyme in ketone body synthesis—was consistently downregulated following BCG challenge [20,21]. HMGCS2 catalyzes the condensation of acetyl-CoA and acetoacetyl-CoA to form HMG-CoA, a critical step in ketogenesis [41]. Ketone bodies serve as alternative energy sources for the brain and other tissues during metabolic stress and have been shown to exert anti-inflammatory and neuroprotective effects [42]. In neurological contexts, HMGCS2 promotes autophagic clearance of amyloid precursor protein (APP) and Tau protein, suggesting a protective role in Alzheimer’s disease [21,41]. Its suppression in TBM may therefore compromise cellular resilience and exacerbate inflammatory injury.

Given the central role of HMGCS2 in ependymal cell homeostasis, we investigated whether its pharmacological activation could mitigate BCG-induced damage. Sodium butyrate (NaB), a histone deacetylase inhibitor, was found to transcriptionally upregulate Hmgcs2 expression [23,24]. Treatment with NaB significantly attenuated BCG-induced ependymal cell apoptosis and preserved ciliary structure and function. We acknowledge that while NaB treatment restores Hmgcs2 expression and rescues ependymal functional deficits [43], our current data establishes association rather than causation. NaB is a broad-spectrum HDAC inhibitor affecting hundreds of genes simultaneously [1]. Future studies employing Hmgcs2-specific siRNA knockdown in naive ependymal cells (to determine whether Hmgcs2 loss alone reproduces BCG-induced phenotypes) or Hmgcs2 overexpression independent of NaB (to determine whether Hmgcs2 restoration alone rescues these phenotypes) are required to establish causality. These experiments represent a priority for our ongoing investigations.

Sodium butyrate (NaB), a short-chain fatty acid and histone deacetylase inhibitor (HDACi) [44], has demonstrated broad therapeutic potential in various neurological disorders [45]. NaB regulates gene expression in the brain through HDAC inhibition, exerting anti-inflammatory, antioxidant, anti-apoptotic, and neurotrophic effects. In neurodegenerative diseases, NaB has been extensively studied [46]. In Alzheimer‘s disease (AD), NaB recovers memory function in 5xFAD mouse models by restoring hippocampal histone acetylation [47], and in Aβ_42_-treated SH-SY5Y neurons, it significantly improves cell viability, attenuates apoptosis, and upregulates neuroprotective and antioxidant genes including BDNF, Nrf2, and SIRT1 [48]. In Parkinson’s disease (PD), systematic reviews and meta-analyses indicate that NaB improves motor function and exploratory behavior in animal models-7 [49], with mechanisms involving KATP channel activation to suppress microglial inflammatory responses [50] and modulation of the gut microbiota to inhibit gut–brain axis inflammation [51]. NaB also shows therapeutic promise in Huntington‘s disease (HD) as an HDACi. In acute brain injury, NaB has demonstrated notable efficacy. In ischemic stroke models, NaB converts pro-inflammatory M1 microglia to the anti-inflammatory M2 phenotype [52] and modulates the TLR4/MyD88/NF-κB pathway to alleviate neuroinflammation [53], significantly reducing cerebral infarct volume [54]. In cardiac arrest-induced brain injury models, NaB improves neurological scores and reduces neuronal apoptosis through similar mechanisms involving microglial polarization and the gut–brain axis [55]. Furthermore, NaB shows potential therapeutic value in amyotrophic lateral sclerosis (ALS) [56], epilepsy [57], and psychiatric disorders including depression and anxiety [58,59]. Notably, a common pathological feature across many of these diseases is microglia-mediated neuroinflammation, in which NaB’s anti-neuroinflammatory effects play a central role [60]. The present study extends the protective effects of NaB to ependymal cells in the context of TBM and identifies Hmgcs2 as a novel downstream target—namely, NaB epigenetically upregulates Hmgcs2 expression, restoring the metabolic adaptability of ependymal cells, thereby preserving ciliary structure and inhibiting apoptosis. This discovery not only enriches the mechanistic landscape of NaB‘s neuroprotective actions but also provides a new therapeutic strategy for adjunctive TBM treatment.

Several limitations of this study should be acknowledged. First, the BCG model, while useful, does not fully recapitulate the virulence and immune evasion mechanisms of Mycobacterium tuberculosis, limiting direct extrapolation to human TBM. Second, it is worth emphasizing that although this study has fully verified that NaB protects the structural and functional integrity of ependymal cells by upregulating Hmgcs2 in vitro, there is still a lack of direct therapeutic evidence of NaB in in vivo models of TBM, which is one of the main limitations of this study. As a short-chain fatty acid, NaB (sodium butyrate) is easy to be rapidly metabolized and cleared in vivo, its efficiency in crossing the blood–brain barrier and the optimal drug delivery strategy still need to be determined by systematic pharmacokinetic–pharmacodynamic (PK/PD) pre-experiments [61]. We did not validate the effect of Hmgcs2 modulation or NaB treatment in an in vivo TBM model, leaving open questions about their systemic efficacy and safety. Third, the precise mechanisms through which Hmgcs2 influences ciliary stability and apoptosis—and how BCG suppresses its expression—remain unclear and warrant further investigation. Finally, the use of a microglial cell line rather than primary microglia may oversimplify cellular responses.

In addition, the public single-cell dataset (GSE199649) used for the integrated analysis in this study [17] has certain limitations. This original study did not explicitly report the absolute number of ependymal cells, which is a rare cell population (typically <1%) in CNS single-cell sequencing, and their insufficient number may affect the statistical robustness of the differential expression analysis. Therefore, we positioned this meta-analysis as supportive circumstantial evidence of the in vitro findings rather than as independent validation. Despite this limitation, our in vitro validation of Hmgcs2 downregulation by direct BCG stimulation of primary ependymal cells (Figure 6A,B) and functional data on NaB protection of ciliary structure and inhibition of apoptosis (Figure 6D–F), both independent of this single-cell dataset, constitute the core chain of evidence for the findings of this study. Future studies using single-cell datasets containing definitive ependymal cell counts or spatial transcriptomics techniques are needed to verify transcriptional changes in ependymal cells in situ in vivo.

Despite these limitations, our study unveils a novel microglia–ependymal immuno-metabolic axis in TBM and identifies NaB as a promising therapeutic agent capable of preserving ependymal function via Hmgcs2 activation. Future studies should explore the in vivo efficacy of NaB, elucidate downstream mechanisms of Hmgcs2, and validate these findings in clinical specimens or models employing fully virulent mycobacteria. These efforts may ultimately contribute to new adjunctive therapies aimed at reducing the morbidity and mortality of TBM.

## 5. Conclusions

This study demonstrates that microglia potently amplify BCG-induced transcriptional dysregulation in ependymal cells during TBM, leading to immune activation and functional impairment. We identify Hmgcs2 downregulation as a central event in ependymal injury and show that sodium butyrate restores Hmgcs2 expression, preserves ciliary integrity, and reduces apoptosis. These findings reveal a key neuroimmune–metabolic axis in TBM and highlight Hmgcs2 as a promising therapeutic target. Further studies should validate these mechanisms in vivo and explore translational applications.

## Data Availability

The single-cell RNA sequencing data reanalyzed in this study are available in the Gene Expression Omnibus (GEO) repository under accession number GSE199649. The datasets generated and/or analyzed during the current study are available from the corresponding author on reasonable request.

## Supplementary Materials

The following supporting information can be downloaded at: https://www.mdpi.com/article/10.3390/arm94040054/s1, Table S1. Primer sequences used in this study; Table S2. BCG+Epen, BCG+BV2+Epen, and single-cell RNA sequencing data.
